# Bacterial Community Composition of Size-Fractioned Aggregates within the Phycosphere of Cyanobacterial Blooms in a Eutrophic Freshwater Lake

**DOI:** 10.1371/journal.pone.0102879

**Published:** 2014-08-21

**Authors:** Haiyuan Cai, Helong Jiang, Lee R. Krumholz, Zhen Yang

**Affiliations:** 1 State Key Laboratory of Lake Science and Environment, Nanjing Institute of Geography and Limnology, Chinese Academy of Sciences, Nanjing, China; 2 Department of Botany and Microbiology, University of Oklahoma, Norman, Oklahoma, United States of America; University of New South Wales, Australia

## Abstract

Bacterial community composition of different sized aggregates within the *Microcystis* cyanobacterial phycosphere were determined during summer and fall in Lake Taihu, a eutrophic lake in eastern China. Bloom samples taken in August and September represent healthy bloom biomass, whereas samples from October represent decomposing bloom biomass. To improve our understanding of the complex interior structure in the phycosphere, bloom samples were separated into large (>100 µm), medium (10–100 µm) and small (0.2–10 µm) size aggregates. Species richness and library coverage indicated that pyrosequencing recovered a large bacterial diversity. The community of each size aggregate was highly organized, indicating highly specific conditions within the *Microcystis* phycosphere. While the communities of medium and small-size aggregates clustered together in August and September samples, large- and medium-size aggregate communities in the October sample were grouped together and distinct from small-size aggregate community. Pronounced changes in the absolute and relative percentages of the dominant genus from the two most important phyla *Proteobacteria* and *Bacteroidetes* were observed among the various size aggregates. Bacterial species on large and small-size aggregates likely have the ability to degrade high and low molecular weight compounds, respectively. Thus, there exists a spatial differentiation of bacterial taxa within the phycosphere, possibly operating in sequence and synergy to catalyze the turnover of complex organic matters.

## Introduction

Due to climate change and anthropogenic carbon and nitrogen runoff, cyanobacterial blooms are becoming more common in freshwater lakes and estuaries throughout the world and threaten the sustainability of aquatic ecosystems [1]. The formation of large mucilaginous cyanobacterial blooms in freshwater lakes restricts light penetration, depleting oxygen levels, thereby reducing water quality adversely affecting the ecosystem [1]. These changes can result in reduction in the numbers of submerged plants, killing of aquatic animals, and alteration in food web dynamics [2]. Furthermore, massive cyanobacterial blooms in eutrophic lakes are dominated by *Microcystis* spp., which produces toxic microcystins that can prevent water consumption [3].

In order to better understand the process and mechanism of cyanobacterial bloom formation, previous studies usually focused on cyanobacterial species composition and chemical and physical factors influencing cyanobacterial growth [4]. However, numerous heterotrophic bacteria were found to be associated with cyanobacteria, and had an important impact on cyanobacterial growth. In fact, cyanobacterial-heterotrophic bacterial associations are commonly observed both inside cyanobacterial colonies/aggregates and within excellular polymers outside cyanobacterial cell walls. Collectively, these microhabitats constitute the cyanobacterial phycosphere. In the phycosphere, bacteria can live freely, attached to the algal surface, or extracellular products [5].

The phycosphere was a niche that might provide a suitable microenvironment for a diverse subset of bacteria [6]. The cyanobacteria excreted an abundance of extracellular organic matter that likely provides energy for associated bacteria [7]. In turn, bacterial partners may play a role in providing CO_2_, nitrogen, phosphorus, sulfur and trace elements to the cyanobacteria [8]. Thus, investigation of microbial communities in the phycosphere of cyanobacterial blooms may help us to understanding why cyanobacteria often dominate phytoplankton communities in eutrophic freshwater ecosystems.

While the microbial community associated with cyanobacterial aggregates/colonies has been widely investigated in recent years [9]–[13], few studies investigated the complex interior structure in the phycosphere of cyanobacterial blooms. In eutrophic lakes and estuaries, cyanobacterial *Microcystis* colonies usually aggregate, and then form mucilaginous *Microcystis* blooms through coagulation of extracellular polymeric substances [14], moreover, cyanobacterial debris and other particle organic matter also was released from the *Microcystis* colonies. As a result, there exist microbial aggregates of various sizes in cyanobacterial blooms due to of nutrient availability [15]. Notably, distinct microcystin production and genotype compositions among size-fractionated *Microcystis* aggregates were observed [15], emphasizing the need to partition aggregates by size for microbial community analysis.

In addition, the non-cyanobacterial community associated with blooms has been intensively studied by the application of various molecular methods, including polymerase chain reaction-denaturing gradient gel electrophoresis (PCR-DGGE) [13] and terminal restriction fragment length polymorphism (T-RFLP) [16]. Because *Microcystis* spp. dominanted bloom samples, the above molecular methods lacked sensitivity and could not accurately reflect the bacterial diversity within cyanobacterial blooms. In comparison, parallel 454 pyrosequencing is a high-throughput analytical method that can generate much more information on community compostion [17]. This technology has been used widely to analyze the microbial community in various environmental samples, but has not been previously used to study cyanobacterial bloom communities.

The objective of the present study was to describe and compare the phylogenetic diversity of the microbial communities in various size-fractioned aggregates within the phycosphere of cyanobacterial blooms. In this study, *Microcystis* blooms in the eutrophic Lake Taihu, were taken from on three dates (10^th^ August, 9^th^ September and 11^th^ October 2012). The bloom samples were filtered to separate communities into three fractions (>100 µm, 10–100 µm, and 0.2–10 µm). Bacterial community composition in the bloom phycosphere was characterized using high throughput sequencing and a well-established β-diversity analytical tool. This study revealed the complex bacterial communities of the phycosphere within *Microcystis* blooms, and suggests possible ecological roles in catalyzing the turnover of complex organic matter released from the cyanobacterial aggregates. These diversity analyses will facilitate current understanding of the distribution and the ecological roles of bacterial communities associated with *Microcystis* blooms in eutrophic lakes.

## Materials and Methods

### Ethics statement

No specific permits were required for the described field studies. The location studied is not privately-owned or protected in any way and our studies did not involve any endangered or protected species.

### Sample collection

Lake Taihu, located in the Changjiang (Yangtze) River delta in eastern China, is a large shallow, eutrophic and temperate lake. The lake has a surface area of 2338 km^2^, and an average depth of 1.9 m. *Microcystis* spp. blooms have been observed in Lake Taihu since the 1990s, usually between June and October. When cyanobacterial blooms occur in Lake Taihu, large *Microcystis* biomass accumulates on and below the water surface.

*Microcystis* bloom samples were collected from the surface water of Meiliang Bay (site A: 31°30′N, 120°11′E; site B: 31°44′N, 120°18′E) within Lake Taihu on 10^th^ August, 9^th^ September, and 11^th^ October 2012. Equal volume bloom samples (5 L) taken from site A and B were mixed. Physiochemical parameters were determined *in situ* Yellow Spring Instruments (YSI, 6600, USA). Total nitrogen (TN) and total phosphorus (TP) were analyzed according to standard methods [18]. Chlorophyll a (Chl a) was determined according to Asai and colleagues [19]. Bloom samples were retrieved by dipping a sterile beaker off the side of a boat from the surface down to a depth of about 5 cm. In August and September, *Microcystis* blooms were driven by the wind to accumulate in Meiliang Bay, forming a dense layer (nearly 20 cm in thickness). However, thin (less than 5 cm in thickness) and brown-yellow bloom layers formed in October, due to lower water temperature. *Microcystis* blooms in August and September were mostly intact and green. However, in October, they were broken and brown-yellow, indicating that cyanobacterial aggregates was decomposing. Therefore, the two blooms samples taken in August and September, represented healthy cyanobacterial bloom biomass and the sample taken in October represented decomposing cyanobacterial bloom biomass.

The phycosphere sample was obtained by taking advantage of the relative buoyancy of the cyanobacterial aggregates. After transferring to the laboratory within a few hours, bloom samples were put into in 50 mL sterile centrifuge tubes, and the tubes were left at room temperature for 2 hours. This process resulted in a layer of the concentrated cyanobacterial aggregates at the top surface of the centrifuge tube. This top layer was regarded as the phycosphere sample of cyanobacterial blooms.

The phycosphere sample was subsequently filtered through a sterile 100 µm nylon net filter (Millipore), and sterile PBS was used to rinse the filter. The biomass retaining on the 100 µm filter was used as large-size cyanobacterial aggregates (named as BCA). The filtrate was then filtered through a 10.0-µm pore-size filter (Millipore, 47 mm diameter), and the biomass on the filter paper was regarded as medium-size cyanobacterial aggregates (named as MCA). The remaining filtrate was further filtered onto a 0.2 µm pore-size polycarbonate filter (Millipore, GTTP, 47 mm diameter), and the biomass on this kind of filter paper included small-size aggregates and free-living cells (named as SC) within the cyanobacterial phycosphere.

Sample biomass was stored at −20°C prior to DNA extraction. For simplification, the samples (BCA, MCA, and SC) in cyanobacterial blooms taken in August, September and October were named as 08BCA, 08MCA, 08SC, 09BCA, 09MCA, 09SC, 10BCA, 10MCA, and 10SC, respectively.

### Nucleic acid extraction and 454 pyrosequencing

Bacterial genome DNA was extracted using two methods in parallel: one using an UltraClean Soil DNA Isolation Kit (MoBio Laboratories, Carlsbad, CA, USA) according to the manufacturer's directions, and another one using a phenol-chloroform protocol as previously described [20]. DNA concentration and purity were then determined using a Nanodrop ND-2000 UV-Vis spectrophotometer (Nanodrop, Wilmington, DE, USA). Purified DNA extracts were stored at −20°C until use. In order to minimize putative bias due to the DNA extraction methods, the DNA obtained by both methods were pooled together in equal concentrations. 16S rRNA genes were amplified using 341F [21] and 907R [22] primers. The PCR amplifications, performed in triplicate for each DNA extract, and a smaller number of PCR cycles were employed in this study. The thermo cycling steps were as follows: 95°C for 4 min, followed by 25 cycles at 95°C for 30 s, 55°C for 30 s, 72°C for 30 s and a final extension step at 72°C for 5 min. The amplicons were quantified by fluorimetry with PicoGreen dsDNA quantitation kit (Invitrogen, Life Technologies, Carlsbad, CA,) and pooled at equimolar concentrations. Roche GS-FLX 454 pyrosequencing was conducted by Meiji Biotechnology Company (Shanghai, China). Pyrosequencing sequence data from this study were submitted to the NCBI Sequence Read Archive (SRA) under accession number SRP042642.

### Bacterial 16S rRNA data processing and analysis

All 16S rRNA pyrosequencing reads were analyzed using QIIME (Quantitative Insights Into Microbial Ecology) [23] software package version 1.6.0 and MOTHUR version 1.30.0 [24]. Sequences were quality controlled using the Split_Libraries.py script with default settings (minimum length 200, maximum length 1000, minimum mean quality score 25, maximum ambiguous bases 0, maximum homopolymer length 6, maximum primer mismatch 0). Pyrosequencing noise was removed using the scripts “denoise_wrapper.py” and “inflate_denoiser.py” in QIIME. After implementation of these quality-control criteria, 55,640 sequences were retained for further analysis.

### Operational taxonomic unit (OTU) assignments and community analysis

The unique.seqs command implemented in MOTHUR version 1.30.0 (24) was used to obtain a non-redundant set of sequences from the high-quality reads. The resulting unique sequences were aligned by align.seqs command against a Greengenes template alignment(http://greengenes.lbl.gov/Download/Sequence_Data/Fasta_data_files/core_set_aligned.fasta.imputed). Aligned sequences were then filtered by filter.seqs command to remove columns that corresponded to ‘.’ or ‘-’ (gaps) in all sequences. The dist.seqs command then calculated uncorrected pairwise distances between aligned DNA sequences. The pairwise distances matrices served as input for Bin.seqs in order to cluster the sequences into OTUs of a defined sequence identity. All OTUs defined at a 0.03 cut-off were classified by classify.otus commands, and a matrix of the OTU abundances for each sample was generated by count.seqs commands. For each OTU, a sequence was chosen as being representative using get.oturep command. Representative sequences were classified by RDP Classifier (http://rdp.cme.msu.edu/classifier/classifier.jsp).

Library coverage, richness and diversity estimates (ACE, Chao1 and Shannon) were calculated for the nine samples using MOTHUR's summary.single commands at a 0.03 and 0.05 distance cut-off Sequences. Rarefaction curves were calculated using rarefaction.single.

In order to graphically represent the structure of a bacterial community, Pareto–Lorenz (PL) evenness curves were constructed, based on the OTU profiles of each library at a 97% sequence identity cut-off level, as previously described [25]. For each library, the OTUs were ranked from high to low based on their abundances. Then, the cumulative proportion of OTUs was used as *x*-axis, and their respective cumulative proportions of abundances on the *y*-axis. The curves were numerically interpreted by the functional organization index (*Fo*), given by the horizontal y-axis projection on the intercept with the vertical 20% x-axis line [25]. The more the PL curve deviates from the 45° diagonal line (the theoretical perfect evenness line), the less evenness can be observed in the structure of the studied community [25].

To compare and perform statistics across samples, the samples were first normalized down to the sample with the fewest sequences using normalize.shared, then calculated using tree.shared in MOTHUR and a tree were generated that describes the dissimilarity among samples. To further describe the community dissimilarity among samples, the Yue and Clayton theta statistic was estimated via the dist.shared command. The resulting distance matrix was then calculated by pcoa command in MOTHUR and visualized in a principle components ordination plot using the first two axes.

## Results and Discussion

### Environmental Conditions

Biologically available nutrients in August were more abundant than in September or October, consistent with the highest primary production, indicated by chlorophyll a levels, occurring in August (Table 1).

**Table 1:** Environmental variables at the three sampling times.

Time	Temp (°C)	pH	TN(mg/L)	TP (mg/L)	Chla(µg/L)
August	31	8.31	9.53	0.64	102.06
September	21	8.22	1.68	0.14	27.91
October	18	8.24	1.34	0.13	16.92

### Diversity of microbial communities

**Figure 1:**
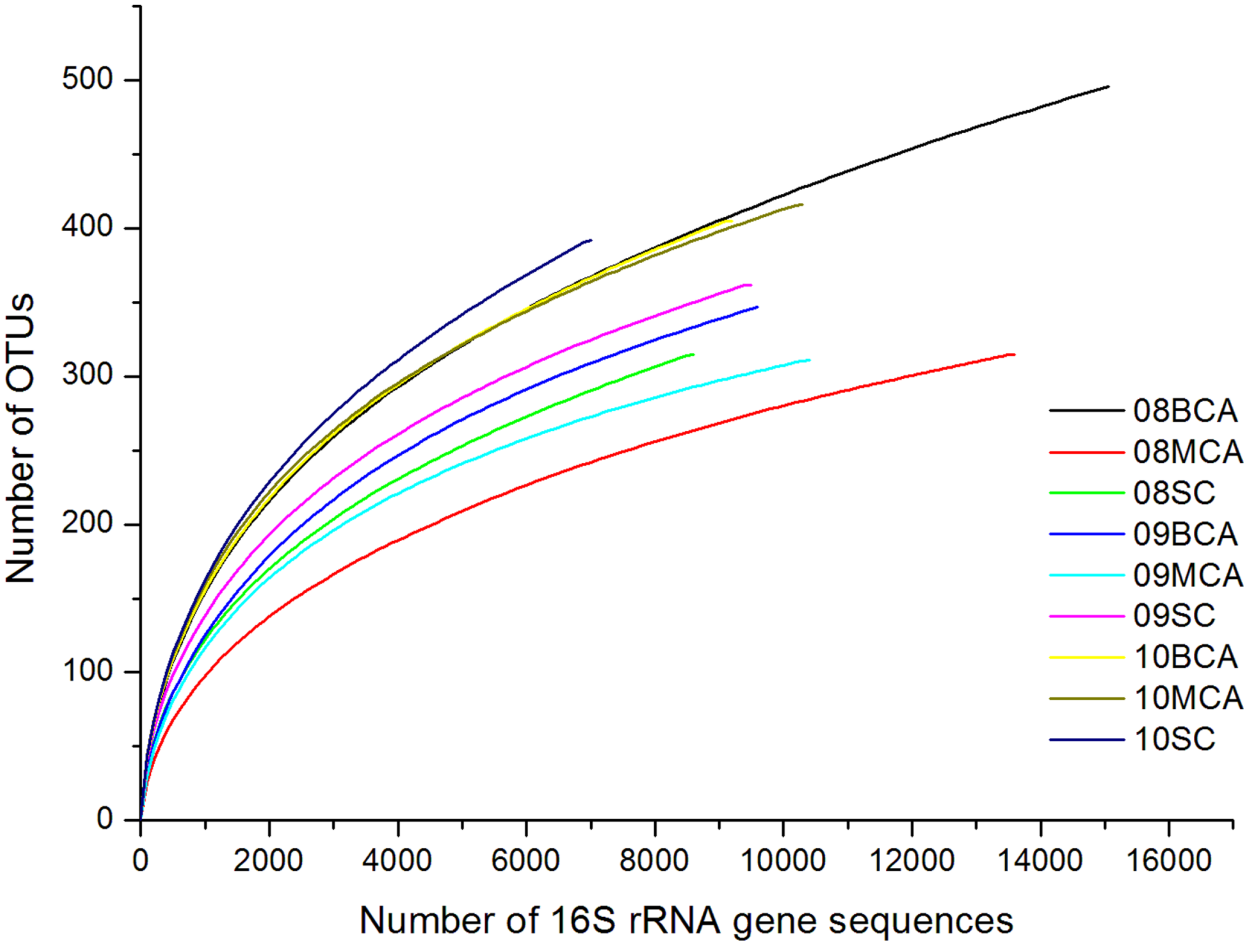
Rarefaction analysis of the 16S rRNA gene sequences among phycosphere samples using an evolutionary distance threshold of 3% (i.e., 97% similarity).

The quality-filtering process removed low quality raw sequence reads, leaving 92,795 high-quality target tags. The average read length was improved to about 494 bp, and the number of reads per sample ranged from 6,951 to 15,055 (Table 2). By performing the alignment at a uniform length of 450 bp, OTUs were clustered at 3% and 5% distances. As species richness increases with the number of sequences in a given sample, all the samples were subsampled using MOTHUR randomly to the same size based on the sample with the smallest sequences number. Coverage analyses showed that the cyanobacterial bloom libraries contained at least 97.6% of the total number OTUs that exist in samples (Table 2). The rarefaction curves appear to almost reach the saturation level (Fig. 1). Hence the 454 pyrosequencing libraries provide us with a nearly complete inventory of the bacterial 16S rRNA sequences present in the samples. On the basis of Richness and Shannon's index, diversity in BCA samples was higher than that in the MCA samples, indicated that *Microcystis* containing aggregates harbored a more diverse bacterial community than that in other organic particles. This may be due to the fact that freshly formed, labile extracellular organic matter is able to sustain a highly diverse community.

**Table 2:** Observed bacterial richness and diversity estimates based on 97% and 95% OTU clusters respectively.

Community	No. Sequences	Cyanobacterial Percentage	No. OTUs		Coverage (%)		Richness (ACE)		Richness (Chaol)	
			97%	95%	97%	95%	97%	95%	97%	95%
08BCA	15055	68.5	496	446	98.7	98.8	927	834	821	758
08MCA	13529	4.2	315	275	99.1	99.2	559	511	480	437
08SC	8561	1.2	315	278	98.5	98.7	604	505	528	431
09BCA	9600	42.8	347	312	98.7	98.8	475	431	483	460
09MCA	10354	5.9	311	279	98.8	99.0	408	370	421	382
09SC	9246	1.3	361	327	98.6	98.7	509	463	540	501
10BCA	9105	27.3	404	360	98.3	98.4	685	629	665	602
10MCA	10214	12.4	416	361	98.5	98.8	582	493	633	511
10SC	6951	0.8	392	343	97.6	97.9	754	652	762	595

Cyanobacterial reads were abundant in the BCA and MCA samples, and were detected at lower percentages in the smaller aggregates (less than 1.5% in the SC samples) (Table 2). More than 99% of the cyanobacterial reads were classified as *Microcystis* spp., with the others assigned to GpVI and GpIIa. The percentage of cyanobacterial reads in the 08BCA sample was the highest at 68.5%, while the cyanobacterial percentage in 10BCA decreased to 27.3%. The cyanobacterial percentage in 10MCA was the highest among the medium sized aggregates at 12.4% compared to 4.2% in 08MCA and 5.9% in 09MCA samples.

### Functional organization analysis

**Figure 2:**
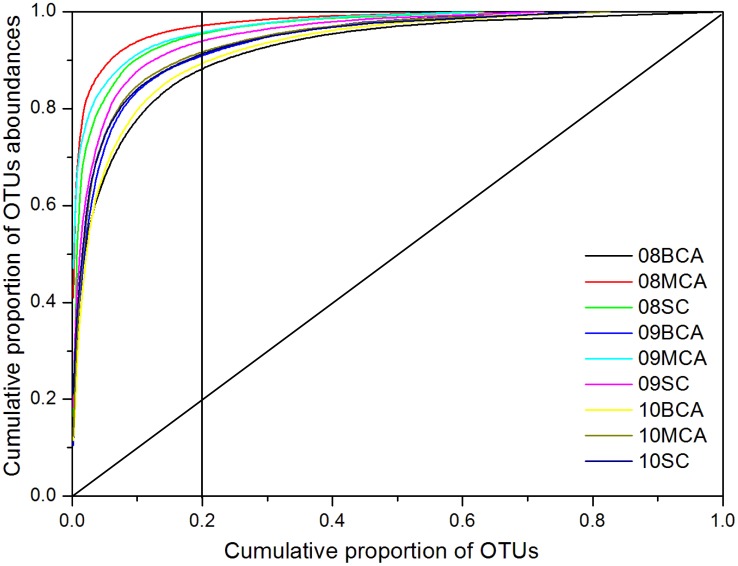
Pareto-Lorenz curves derived from phycosphere samples in Lake Taihu. The 16S rRNA gene sequences were divided in OTUs based on a sequence similarity threshold of 97% and the OTUs were ranked from high to low, based on their abundance. The Pareto-Lorenz evenness curve is the plot of the cumulative proportion of OTU abundance (y-axis) against the cumulative proportion of OTUs (x-axis). The *Fo* index, i.e. the combined relative abundance of 20% of the OTUs, is shown. The 45° diagonal is the Pareto-Lorenz curve of a community with perfect evenness.

Pareto–Lorenz curve distribution patterns were plotted after the cyanobacterial OTUs were excluded for all samples (Fig. 2). When all samples were grouped together, 40.8% of sequences were assigned to the top 10 most abundant OTUs and this number increased to 85.3% for the top 100 most abundant OTUs. For individual samples, 20% of the OTUs contained 85 to 95% (on average 91%) of the cumulative sequence abundance. The latter number is the *Fo* index, which when higher than 85% represents a specialized community in which a small amount of the species is dominant and all the others are present in low numbers [25]. Thus, all these communities were highly specialized, and as there were major differences among communities, we conclude that they were affected by the phycosphere conditions created by cyanobacteria. Others have also observed the selection of specific bacterial species within the Microcystis phycosphere by comparing *Microcycstis*-attached bacteria (phycosphere bacteria) with other microbial communities living in the same aquatic ecosystem [11], [13].

### Similarity analysis

**Figure 3:**
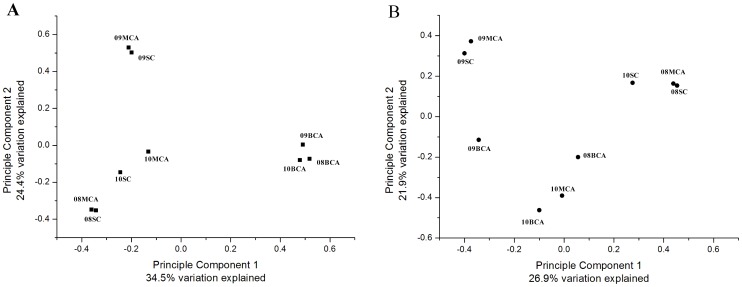
Principle component analysis of the phycosphere samples including cyanobacterial reads (A) and excluding cyanobacterial reads (B). The Yue and Clayton measure of dissimilarity between the structures of the communities was estimated and visualized using the dist.shared and pcoa commands of Mother.

The abundance and diversity of OTUs in our samples were compared using multivariate ordinations (Fig. 3). When all cyanobacterial reads were included, BCA communities at all three sampling times clustered together and separated from other two size fraction communities (Fig. 3a). This may be due to the high relative abundance of *Microcystis* spp. in the large aggregates (Table 2). Thus, *Microcystis* sequences were excluded in order to compare non-cyanobacterial bacterial community composition within the blooms. When all cyanobacterial reads were excluded, BCAs were much less related to each other (Fig. 3b), indicating that large aggregate communities differed over the sampling times. In addition, 08MCA and 08SC clustered together and were separate from 08BCA and 09MCA and 09SC clustered together and were separate from 09BCA. However, 10BCA clustered together with 10MCA and both were separate from 10SC.

Associated bacteria within the cyanobacterial phycosphere are strongly affected by the physiological status of cyanobacteria inside [26]. Thus, the dominance of *Microcystis* cells and microenvironments created within *Microcystis* aggregates were likely the major contributing parameter for the spatial variation observed in the bacterial community structure. *Microcystis* blooms in August and September were green, healthy and intact, whereas in October, they were yellow and broken up, likely as a result of low water temperature and lack of nutrient availability (Table 1). The overall abundance of cyanobacterial cells in BCA in August and September then led to distinct bacterial communities in BCA compared to MCA and SC. In comparison, for cyanobacterial bloom biomass taken in October, the cyanobacterial percentage in BCA was only around two fold of that in MCA. It seems likely that MCA in October were derived from the decomposition of BCA. As a result, in October, there was a similarity between the bacterial community in BCA and MCA.

### General bacterial composition

**Figure 4:**
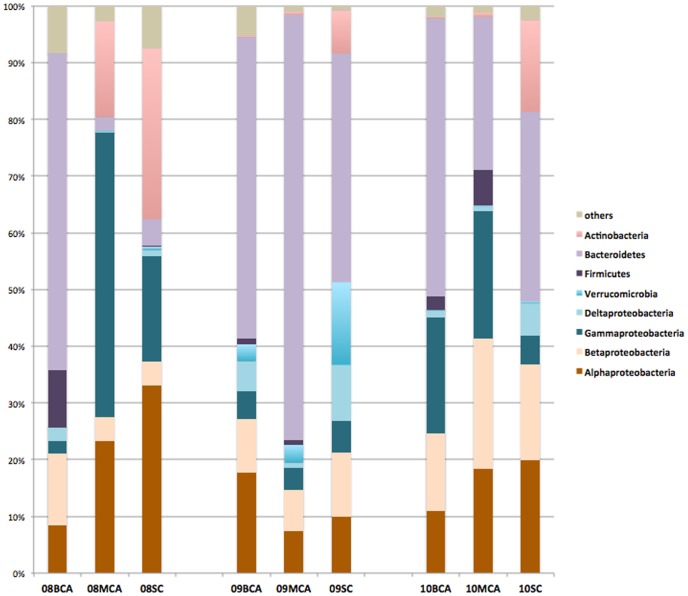
Relative abundance and bacterial composition obtained by pyrosequencing from phycosphere samples in September and October, by phylum. Phylogenetic classification for the pyrosequencing analysis obtained from Ribosomal Database Project Classifier analyses.

All of our non-cyanobacterial sequences were affiliated with at least 22 bacterial divisions: *Proteobacteria*, *Bacteroidetes*, *Actinobacteria*, *Firmicutes*, *Verrucomicrobia*, *Acidobacteria*, *Armatimonadetes*, *Chlamydiae*, *Chlorobi*, *Chloroflexi*, *Fibrobacteres*, *Firmicutes*, *Fusobacteria*, *Gemmatimonadetes*, *Lentisphaerae*, *Nitrospirae*, *Planctomycetes*, *Spirochaetes*, *Verrucomicrobia* and Candidate divisions OD1, SR1 and TM6. The majority of phycosphere sequences belonged to the five major phyla: *Bacteroidetes*, *Proteobacteria*, *Actinobacteria*, *Firmicutes* and *Verrucomicrobia*, and these major groups varied in relative abundance among the samples (Fig. 4).

Of these major phyla, *Bacteroidetes* was the most abundant phylum in all samples, accounting for 2.4–74% of total non-cyanobacterial sequences (Fig. 4). This is in line with other studies [27]–[28] and thus indicates that members of the clade appear to be particularly adapted to bloom conditions, most likely as a result of their ability to degrade complex bio-macromolecules [29] and alga-derived metabolites [27]. *Proteobacteria* was also abundant and dominated 08MCA with a relative abundance of 77%. Other studies have shown *Proteobacteria* as a significant fraction of bloom associated bacteria [9], [12], [13]. *Alpha-*, *Beta-*, *Gamma-*, and *Deltaproteobacteria* were all detected in all nine samples, but relative abundances varied. *Alphaproteobacteria* was the dominant class in the 08SC (32% of the total). *Actinobacteria* was the third most abundant phylum and dominated in the SC libraries, but was rarely detected in the BCA samples. *Actinobacteria* have been previously shown to be the most abundant phylum in 16S rRNA clone libraries prepared from bacterioplankton communities during the period of cyanobacterial bloom in Lake Taihu [30]. In contrast, the phylum *Firmicutes* was most abundant in the BCA and MCA samples rather than in the SC samples. The phylum *Verrucomicrobia* was abundant in September and its relative abundance was as high as 14% in the 09SC samples. However, sequences belonging to *Verrucomicrobia* were rare in August and October.

### Comparative bacterial composition of different sized aggregates at genus level

To further compare the bacterial composition within the *Microcystis* phycosphere,, bacterial composition determined at genus level and those with a relative percent greater than 1% of the sample reads are shown (Table 3). With the exception of 08MCA, 08SC and 10SC, *Flavobacterium* sp. was most abundant in all phycosphere samples comprising 20% of all non-cyanbacterial sequences and it was also very unevenly distributed. This genus was most abundant in September with 66% of all sequences in the 09MCA. Previously, *Flavobacteria* were found to be abundant during phytoplankton blooms, particularly during cyanobacterial blooms in freshwater environment [10], [28], [31]. *Flavobacterium* sp. are likely well adapted to the cyanobacterial phycosphere, especially that of *Microcystis*, and may play an important role in degrading the chemically stable cyanobacterial hepatotoxins [32] and enhancing aggregation of *M. aeruginosa* [26].

**Table 3:** The relative abundance of the predominant phylogenetic groups at the genus level.

		Percentage (%)								
Taxa	Genus	08BCA	08MCA	08SC	09BCA	09MCA	09SC	10BCA	10MCA	10SC
***Bacteroidetes***	*Alkaliflexus*	13.16	L	L	L	L	L	3.52	L	L
	*Chryseobacterium*	0.62	L	L	L	L	L	L	2.26	L
	*Flavobacterium*	3.87	0.66	0.15	44.29	66.82	25.48	33.06	21.08	L
	*Sediminibacterium*	L	0.34	0.43	L	L	L	L	L	25.62
	*Solitalea*	0.65	0.23	L	L	4.92	5.54	L	L	L
**Alpha**	*Azospirillum*	L	15.85	1.80	L	0.31	L	L	5.50	L
	*Methylobacterium*	L	L	L	L	L	L	L	L	8.38
	*Pelagibacter*	L	3.58	16.47	L	L	0.65	L	L	4.40
	*Rhodocista*	L	0.19	L	1.23	L	0.33	L	3.13	L
	*Roseomonas*	1.97	0.51	0.63	1.54	1.17	0.73	0.70	0.31	L
**Beta**	*Dechloromonas*	5.45	L	L	L	L	L	0.73	L	L
	*Limnobacter*	L	0.50	L	0.74	1.70	L	1.12	3.37	0.64
	*Methylophilus*	L	0.73	0.49	L	0.51	2.13	L	0.13	4.84
	*Methylovorus*	L	L	L	L	0.31	2.07	L	L	L
	*Propionivibrio*	0.22	L	L	L	L	L	L	5.05	L
	*Vogesella*	L	L	L	0.78	0.29	L	0.97	6.92	1.02
**Gamma**	*Aeromonas*	0.54	2.67	1.01	0.56	1.26	1.92	4.98	12.53	L
	*Legionella*	L	L	L	L	0.52	1.87	L	0.79	L
	*Pseudomonas*	0.17	30.52	15.42	0.94	1.75	0.35	5.28	5.76	0.48
	*Rheinheimera*	0.18	0.34	0.37	0.18	L	L	L	0.79	1.75
**Delta**	*Bdellovibrio*	L	L	L	0.42	L	0.51	L	L	3.85
	*Peredibacter*	L	L	0.21	3.99	0.47	L	L	L	1.06
***Gemmatimonadetes***	*Gemmatimonas*	0.84	0.34	L	4.06	L	L	0.97	L	L
***Firmicutes***	*Clostridium*	0.48	3.21	L	L	0.33	L	2.12	3.56	L
***Verrucomicrobia***	*Opitutus*	L	L	0.21	2.65	0.10	13.74	L	L	L

Relative abundance is defined as the number of sequences affiliated with that taxon divided by the total number of sequences per sample (%). Taxa represented occurred at >1% abundance in at least one sample. “L” represented low abundance (<0.1%).

We observed obligate gram-negative predatory bacteria belonging to the *Bdellovibrio*-and-like organisms (BALOs), which include *Bdellovibrio* and *Peredibacter* (Table 3). These organisms may lyse *Microcystis* releasing the cell contents to the phycosphere. The representative 454 sequences of *Bdellovibrio* are closely related to *B. bacteriovorus* (97% similarity), which is a gram-negative, vibrio-shaped bacterium that preys on other gram-negative bacteria. It was previously reported that *Microcystis* cells were lysed by *Bdellovibrio*-like bacteria through breakdown of cell structures [33]. *Peredibacter* sp. consists of predatory, gram-negative, bacteriovorous organisms that require a gram-negative host as prey to complete a biphasic life cycle. Members of the genus *Peredibacter* are generally regarded as soil-dwellers [34], and were not previously shown to exist with high abundance in cyanobacterial phycosphere.

All phycosphere samples included some obligate or facultative anaerobic microbes (Table 3). For example, September samples were dominated by *Opitutus*, which is an obligatory anaerobic member of the phylum *Verrucomicrobia*, usually inhabiting anoxic environments. *Microcystis* bloom forms a dense scum that can be 10–30 cm in thickness in September, when local temperatures are higher than 30°C. At this time, dissolved oxygen is rapidly exhausted below the surface of the bloom and the microenvironment quickly becomes anoxic [35]. The metabolism of *Opitutus* sp. is suited for growth on plant-derived (poly)saccharides [36]. Thus, *Opitutus* may decompose organic matter released by *Microcystis*, allowing it to be abundant in anaerobic environments created by the cyanobacterial bloom. In August and October samples, *Clostridium* and *Dechloromonas* were abundant. The representative 454 sequences of *Dechloromonas* are closely related to *D. agitata* (98% similarity), which has the ability to reduce (per)chlorate [37]. The relatively high abundance of *Dechloromonas* indicated that there exist anaerobic niches in August phycosphere. *Clostridium* sp. was also observed to be highly abundant, coexisting with *Microcystis* and is likely involved in the hydrolysis of *Microcystis* biomass [38].

Fig. 3B shows that 08MCB and 08SC cluster together and 09MCB and 09SC clustered together. *Azospirillum*, *Pelagibacter*, *Pseudomonas* and ACK-M1 were abundant in the 08MCB and 08SC samples and *Aeromonas*, *Legionella*, *Methylophilus* and *Methylovorus* were abundant in the 09MCB and 09SC (Table 3). These genera seem to have ability to degrade a range of high to low molecular weight (MW) compounds coming from cyanobacterial debris and extracellular complexes. *Pseudomonas* is a widespread genus, made up of versatile aerobic bacteria [39] can utilize various substrates, including a variety of macromolecules such as hydrocarbons and aromatic compounds [40]. *Legionella* strains have been previously observed in cyanobacterial biofilms [41] and are thought to grow on organic compounds produced by the cyanobacteria [42]. *Pelagibacter* had a pronounced preference for glutamine and glutamate over 7 other amino acids *in situ* [43]. In addition, *Pelagibacter* was also able to exploit other monomeric sources of organic carbon including glucose, fructose or acetate [43]. *Methylophilu*s is a restricted facultative methanol-utilizing bacterium and has been observed in high abundance in *Microcystis* blooms [9]. *Methylovorus* is facultatively methylotrophic bacterium [44], but has been rarely reported to be abundant in cyanobacterial bloom samples.

In comparison to MCA and SC samples in August and September, BCA samples from August and September had high levels of high MW organic matter degrading genera including *Alkaliflexus*, *Dechloromonas*, *Clostridium* and *Gemmatimonas*. *Gemmatimonas* belongs to the phylum *Gemmatimonadetes* and is frequently associated with cyanobacterial mats [45].

October samples were also dominated by various organic matter degraders. 10BCA and 10MCA bacteria that have ability to degrade high MW and/or compounds with a complex structure were abundant during October and include *Alkaliflexus*, *Propionivibrio*, *Pseudomonas* and *Clostridium*. The representative 16S sequences of *Alkaliflexus* are closely related to *A.imshenetskii*, which is capable of decomposing plant polymers (xylan and starch), as well as mono- and disaccharides [46]. The representative 16S sequences of *Propionivibrio* were closely related to *P. limicola*, which degrades hydroaromatic compounds [47].

It is important to point out that *Aeromonas* sp. dominated both the 10BCA and 10MCA. A recent study suggested that the *Aeromonas* strains could be responsible for gastrointestinal symptoms declared following recreational exposure to cyanobacterial bloom [48]. The water from Lake Taihu is used as a source of drinking water, and therefore could transfer pathogens like *Aeromonas* during cyanobacterial blooms.

The most abundant genera in 10SC were in the *Pelagibacter*, *Methylobacterium* and *Methylophilus*, which all have a small cell size and the ability to degrade low MW carbon organic compounds. *Methylobacterium* can use methanol and methylamine as well as C2, C3 and C4 compounds to grow [49]. Nearly 10% of non-cyanobacterial reads in 10SC were classified as *Methylobacterium*. Few studies have detected this genus at high abundance in the cyanobacterial phycosphere. The closest relative of the 16S sequence of *Methylobacterium* was *M. hispanicum* (99% similarity). Also, the genus *Methylophilus* that only degrades acetate and single-carbon compounds [50] was detected in 10SC with 8.0% of the reads.

Spatial differentiation of bacterial taxa within the phycosphere was observed in three months bloom samples in Lake Taihu. This variation could be a result of a specific metabolic sequence includes sequential processing and degradation of specific components within extracellular organic matter (EOM) released by cyanobacteria. In previous chemical analyses of bloom samples, high MW and hydrophilic organic compounds accounted for the majority of *Microcystis aeruginosa* EOM which was comprised of protein-like, polysaccharide-like and humic-like substances [51]. Our present findings suggest that BALOs may lyse *Microcystis* cells and then *Flavobacterium*, *Gemmatimonas*, *Aeromonas*, *Pseudomonas* and others, perhaps including anaerobic microbes might metabolize high MW EOM and/or *Microcystis* cell contents to single- or low-carbon organic compounds, and carbon dioxide. The single- or low MW carbon compounds could be further utilized by *Pelagibacter* sp., *Methylobacterium* sp., *Methylophilus* sp. and *Methylovorus* sp. in the terminal portion of this aerobic food chain. Although confirmation awaits further experimentation and chemical analyses, if correct, this metabolic pathway may provide a partial explanation for the ubiquitous presence of methylotrophs in association with cyanobacterial blooms [9], [32].

### Possible ecological role of bacterial communities in different-size aggregates within the phycosphere of cyanobacterial blooms

In this study, it was observed that phylotypes capable of utilizing high molecular weight (HMW) compounds, were mainly present in BCA within the phycosphere of cyanobacterial blooms, while phylotypes capable of utilizing single-carbon or low MW organic compounds, were only present in SC. As one of the most common bloom-forming cyanobacteria, *Microcystis aeruginosa* produces numerous secondary metabolites and EOM during normal growth [14], [51]. Compared to exudates from cyanobacteria, lysates from cyanobacteria consist of more complex organic compounds [52]. These high MW compounds are degraded and recycled by cooperation from a variety of bacterial species [53]. As cyanobacteria are at highest abundance in the BCA phycosphere, attached bacteria in BCA are likely responsible for initial degradation of HMW to lower MW compounds, utilized by bacterial communities in MCA and SC. The varied organic carbon compounds within different-size particles in the cyanobacteria blooms resulted in differentiation of bacterial communities among BCA, MCA and SC.

**Figure 5:**
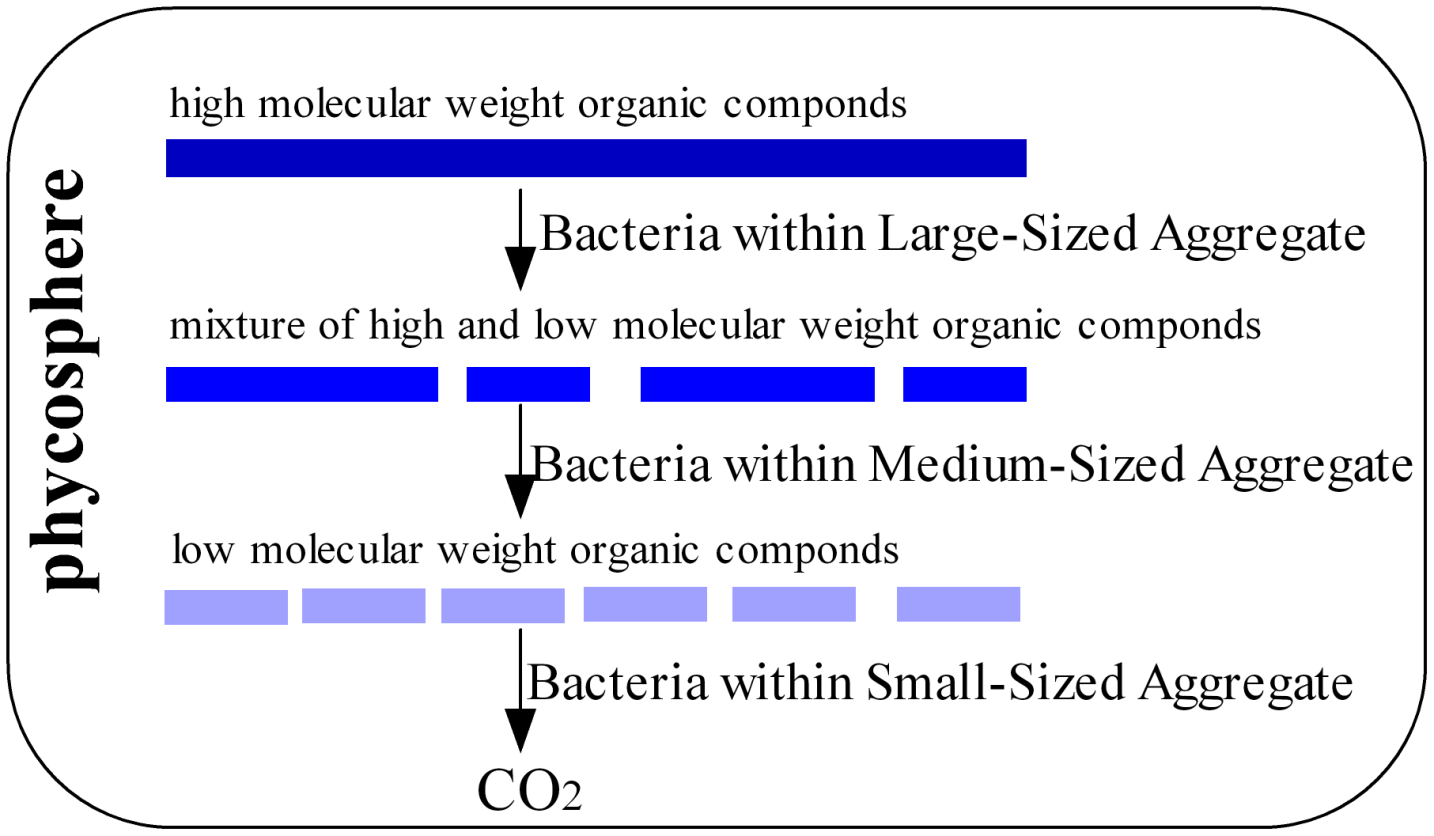
Organic carbon-utilization conceptual model illustrating the role of bacteria on different-sized aggregates in the cyanobacterial phycosphere.

Based on above analyses, we have proposed a conceptual model regarding roles for bacteria in using organic compounds in different-size aggregates within the phycosphere of cyanobacterial blooms. As illustrated (Fig. 5), HMW organic compounds in the phycosphere are degraded by bacterial communities from large-size aggregates resulting in small-size aggregate. Bacterial phylotypes for utilization single-carbon compounds, including *Pelagibacter* sp., *Methylobacterium* sp., *Methylophilus* sp. and *Methylovorus* sp., are present in SC at higher than 2% concentration, indicated that at least a fraction of organic matter within the phycosphere is well utilized. Thus, bacteria within the phycosphere could efficiently provide nutrients and trace elements to the cyanobacteria through recycling the organic matter, which allow persistence of cyanobacterial blooms in freshwater lakes.

### Conclusion

This study separated the phycosphere communities into three fractions and illustrated the complex and highly organized bacterial communities of the phycosphere within *Microcystis* blooms. Functional organization analysis suggested that bacterial composition was highly influenced by phycosphere conditions created during bloom formation, persistence and subsequent decomposition. The bacterial communities on large-to-small size aggregates were able to degrade high-to-low MW compounds, respectively. HMW organic compounds in the phycosphere were degraded in a stepwise manner by bacterial communities from large-size aggregates to small-size aggregates. With the coordinated utilization of complex organic matter, nutrients and trace elements are efficiently recycled within the phycosphere to facilitate the maintenance of cyanobacterial blooms in aquatic environments.
